# Extending the vibroscape to agroecosystems: investigating the influence of abiotic factors and monitoring insect vibrational signaling

**DOI:** 10.7717/peerj.14143

**Published:** 2022-11-17

**Authors:** Imane Akassou, Livia Zapponi, Vincenzo Verrastro, Marco Ciolli, Valerio Mazzoni

**Affiliations:** 1 DICAM Department of Civil, Environmental and Mechanical Engineering, University of Trento, Trento, Italy; 2 Research and Innovation Centre, Fondazione Edmund Mach, San Michele all’Adige, Trento, Italy; 3 CIHEAM–IAMB—International Centre for Advanced Mediterranean Agronomic Studies, Bari, Italy; 4 C3A Centre Agriculture Food Environment, University of Trento, Trento, Italy

**Keywords:** Vibroscape, Vibrational signals, Vineyard, Abiotic factors

## Abstract

Environmental conditions are crucial factors that influence communication systems and affect animal behavior. Research in the field of biotremology has improved our understanding of insect behavior, ecology, and evolution. However, the interactions between vibrational signaling and environmental factors are less studied, mainly because of technical issues faced in field trials. We therefore developed and tested an approach to investigate the effect of abiotic factors on insect vibrational signaling and explored its implementation as a monitoring tool for insect vibrational signals, using a vineyard as an agroecosystem model. Our results showed a significant decrease in insect signaling activity during unsuitable conditions of high temperature and wind velocity. We determined for the first time, the daily signaling pattern of the two insect pests, *Scaphoideus titanus* and *Halyomorpha halys*, in natural conditions. Biotremology techniques could be profitably used to monitor not only the presence of target pest species but also the biodiversity associated with vibrational signaling insects. In particular, the method implemented in this study could be used as a tool to compare the quality of cultivated areas under different management systems.

## Introduction

Animals live in complex ecosystems where sending and receiving information is crucial for reproduction and survival (Dominoni et al., 2020). In the case of acoustic and vibrational communication, environmental conditions, presence of noise, and interactions with their surroundings may directly or indirectly influence the signaling behavior of individuals of a species (Halfwerk et al., 2011; Römer, Lang & Hartbauer, 2010; Trillo et al., 2016). Therefore, it is essential to achieve a comprehensive understanding of the relationship between animal behavior and the environment where animals live in order to determine the influence of changes in environmental conditions on their vibrational communication. The study of soundscape ecology has contributed to develop the discipline of eco-acoustics, which investigates the relationship between the sound and the environment (Sueur & Farina, 2015). This discipline studies the sounds characterizing diverse ecosystems and relies on non-invasive methods to assess, for example, the impact of human activities or derived effects (*i.e.,* climate change) on biodiversity (Desjonquères, Gifford & Linke, 2020; Krause & Farina, 2016; Linke et al., 2018). A similar approach is extendable to biotremology, which studies the production, perception, and transmission of mechanical vibrations through a substrate (Hill & Wessel, 2016). The study of vibroscape, which consists of recording biological, geophysical, and anthropogenic vibrations deriving from a given landscape, is a new crucial opportunity to include a substantial number of animal species that are ubiquitous and use substrate-borne vibrations (Šturm et al., 2021). Unlike bioacoustics, biotremology studies currently require conspicuous and heavy equipment (*i.e.,* laser vibrometer and associated devices), a constant power supply and the presence of personnel on the spot during long recording sessions (Šturm, Polajnar & Virant-Doberlet, 2019). These technical issues have, in the past, made biotremology trials confined to laboratory soundproof chambers and have reduced the possibility to perform recordings outdoor and, consequently, to measure the effect of the environment on the insect vibrational behavior. However, in the last few years the availability of new tools and devices at lower costs and higher/reliable performance has allowed scientists to investigate the vibroscape in natural contexts (Šturm, Polajnar & Virant-Doberlet, 2019; Šturm et al., 2021; Tishechkin, 2013). Another important limiting factor is the almost total absence of libraries of vibrational signals (Šturm, Polajnar & Virant-Doberlet, 2019). Only a few species (often of economic relevance) have been investigated, and furthermore, since each species can be endowed with a large repertoire of signals (*e.g.*, López Díez, 2019; Polajnar et al., 2016b), different signals do not necessarily correspond to different species. In addition, choosing the best moment (time and season) to carry out recordings is crucial in order to optimize the efforts and maximize the efficiency. In fact, the assemblage of multiple individuals and insect species, signaling together, often results in time and space signaling variability (Šturm, Polajnar & Virant-Doberlet, 2019). One driver of this variability for insects resides in abiotic factors, such as wind and temperature, which can impact insect signaling (Gasc et al., 2018; McNett, Luan & Cocroft, 2010). Insects, as ectothermic animals, are largely affected by environmental temperature that can have positive or negative impact on their biological and physiological processes (Colinet et al., 2015). Therefore, studying insect behavior in natural conditions can improve the understanding and interpretation of their signals (*e.g.*, Kerchev, 2020).

Agroecosystems, as compared to other ecosystems, are usually characterized by relatively low biodiversity and are dominated by one or few specialized species (*e.g.*, pests) (Samways, 2005; Sisterson, Dwyer & Uchima, 2020) with the occurrence of some generalists and/or occasional species. Because of this simplification, a vibroscape investigation conducted in an agroecosystem would be less complicated than in a natural environment and could also provide relevant information about the presence, abundance, and phenology of certain species typical of that crop. In this research study, we chose an organic vineyard in northern Italy as a model habitat which includes insects that primarily communicate by substrate-borne vibrations. Among these, the leafhoppers (Hemiptera, Cicadellidae) *Scaphoideus titanus* Ball, 1932 and *Empoasca vitis* Göethe, 1875, two major grapevine pests (Mazzoni et al., 2009; Nieri & Mazzoni, 2018), whose vibrational communication has been described in detail (Nieri & Mazzoni, 2018; Polajnar et al., 2016b), have the possibility to be reliably detected through vibroscape analysis. Our aims were thus to examine how the vibroscape biological component might be affected by diel activity patterns and abiotic factors and whether the adopted approach has the potential to be implemented as a monitoring tool for key pest species of insects in the vineyard. We expect variability in their vibrational signaling according to the diel changes in temperature and wind velocity. In particular, as shown in other studies (*e.g.*, Ahmed et al., 2016; Conrad, Stöcker & Ayasse, 2017; Long et al., 2012), vibrational signaling should increase at optimal temperature and decrease with high wind velocity. Investigating such variability can contribute to a better planning/scheduling of further vibroscape studies as well as insect monitoring in an agroecosystem.

## Materials and methods

### Recording and analyses of vibrational signals

#### Recording of vibrational signals in the vineyard

Trials were conducted from the beginning of July to the end of August 2019, in an organic vineyard of 0.28 ha on the campus of Fondazione Edmund Mach (San Michele all‘Adige, Trentino, Italy; 46.18953 N, 11.13625 E, WGS84). The vineyard was limited by a wooded area (main plant species: *Carpinus betulus*, *Alnus glutinosa*, *Crataegus monogyna* and *Quercus pubescens*) on the east, a country road bordered by a ditch on the west, and vineyards on the north and south sides. Recording sites were chosen randomly in the vineyard, the distance between neighboring sites ranged between 6–18 m (see Fig. S1). To record the insect vibrational signaling throughout the day, two continuous sessions were conducted: S1, from 7:00 to 14:00 and S2, from 14:00 to 21:00. Each session was replicated 13 times (26 sessions in total), resulting in a total duration of 173 h of recording. Days with predicted storms and strong wind were avoided while recording sessions with sudden strong wind, which caused frequent movement of the plants, were canceled or terminated 2 to 3 h before ending the 7 h duration. Data from hourly climate measurements (wind velocity (m s^−1^), air pressure (hPa), relative humidity (%) and air temperature at 2m height (°C)) were obtained from the automated weather station website of Fondazione Edmund Mach in San Michele all’Adige (46.18352 N, 11.12055 E, WGS84; http://meteo.iasma.it/meteo/index.php), which automatically stored data every 10 s and averaged measurements over an hour.

#### Recording of vibrational signals of *S. titanus* in semi-field conditions

This experiment served as a control for the daily vibrational activity of *S. titanus*, to assess the reliability of the method by evaluating the correspondence between field (unknown population density) and semi-field (known population density). We excluded females from the experimental cages, and studied only the signaling behavior of males, because females emit signals only when elicited to respond by male calling songs and the presence of active females can trigger males rivalry behaviors (Mazzoni et al., 2009). The latter were collected, in August 2020, from an infested vineyard near the investigated area and immediately transferred into mesh rearing cages (Bugdorm-6620, 60 × 60 × 120 cm, MegaView Science Co., Ltd., Xitun Dist., Taiwan), in groups of 6 males that were supplied with one year old grapevine plants (*Vitis vinifera* L. cv. Pinot noir grafted on Kobber 5BB) and recorded only once. Five minutes before initiating recordings, the 6 males were collected from the rearing cages and placed on a grapevine plant inside a mesh cage that was positioned outdoors. Similar to the vineyard experiment, trials were performed in two recording sessions (S1: from 7:00 to 14:00 and S2: 14:00 to 21:00). Each session was replicated 6 times (12 sessions in total), resulting in a total duration of 84 h of recording. For uniform environmental conditions, sessions were divided into periods of the day in both experiments: S1 was called “morning” while S2 was divided in “afternoon” (from 14:00 to 18:00) and “evening” (from 18:00 to 21:00).

### Signal recording and analysis

**Figure 1:**
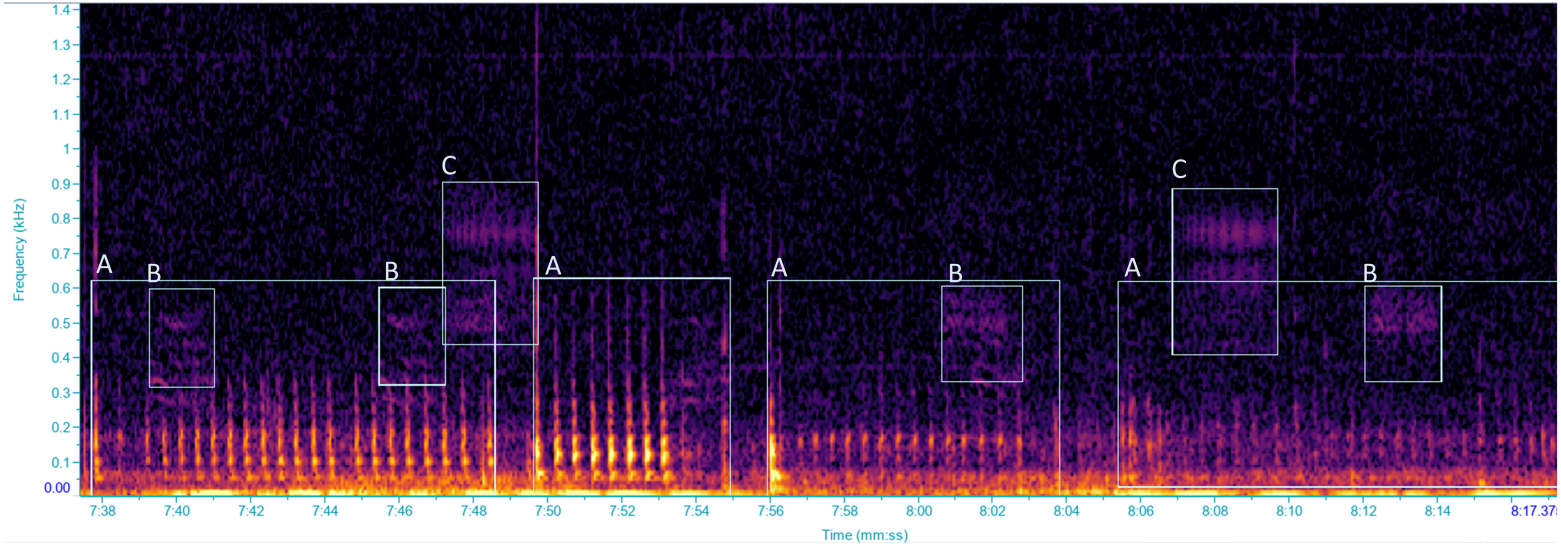
Spectrogram of vibrational signals emitted by: (A) *Scaphoideus titanus*, (B and C) unidentified insects. Selections labelled with the same letters were classified as the same VST. Spectrogram was generated in Raven Pro 1.6 with Hanning window of 1,024 samples with 75% overlap.

Vibrational signals were recorded using a portable laser doppler vibrometer (PDV-100, Polytec GmbH), which measures vibrational velocities. The laser was plugged into a rechargeable battery (PDV-Li, Polytec GmbH) through a charger (PDV-CH, Polytec GmbH). The laser beam was focused onto a reflective sticker, attached to a green stem of the grapevine plant (diameter ca. one cm) in both the vineyard and the semi-field experiments, at approximately 20 cm from the plant stem. Signals were acquired using the software BK Connect (Brüel and Kjær Sound &Vibration A/S, Nærum, 104 Denmark) at a sampling rate of 44.1 kHz and a resolution of 16 bit, through a multichannel calibrated data acquisition device (LAN XI type 3050-B-040, Brüel and Kjær Sound & Vibration A/S, Nærum, Denmark, with 24 bit AD converter, powered by a seal acid battery, MKC12120), and stored on a portable computer hard drive. To accommodate the processing of signals to manageable proportions for a more convenient analysis, recordings were automatically saved as 10-minute wav files. When an insect vibrational signal overlapped two recordings it was considered to be one vibrational signal (however the number of signals was not considered in our study), and the duration of the signal was calculated as the sum of the two pieces. Spectrogram analysis was performed with the software Raven Pro 1.5 (The Cornell Lab of Ornithology, Ithaca, NY, USA) using Fast Fourier Transform, with a 512 samples Hann window and 75% overlap. Each recording was screened, by visual and aural inspection, to characterize the vibrational signal types (VST) which were defined as different types of vibrational signals, classified according to distinct temporal and spectral characteristics (Šturm et al., 2021), focusing on vibrational signals of insects (Fig. 1). Every 10 min recording was screened before zooming into the time and frequency scales depending on the VSTs for precise selections. During recording sessions, the occurrence of audible airborne signals was annotated so as to be excluded during the analysis. Throughout the analysis, vibrational signals of another pest species, *Halyomorpha halys* (Stål, 1855), were identified in our recordings by comparisons with our signal library (Polajnar et al., 2016b), whereas other substrate-borne signals, unidentified, were grouped in different VSTs. Vibrations characterized by the lack of a well-defined spectral/temporal structure and presumably derived by insect mechanical activities (*i.e.,* walking, scratches), as well as those resulted from abiotic and anthropogenic sources and from air-borne sounds (*i.e.,* cicadas or birds) were excluded from the analysis.

### Statistical analyses

Statistical analyses were performed with R version 4.0.2 (RTeam, 2018) run in the R Studio interface (R Studio Team, 2020). Plots and graphic design were done using “ggplot2” and “cowplot” (Wickham, 2016; Wickham & Wilke, 2019). The signaling activity (SA) was calculated as the time (in minutes) insects spent signaling per hour. To better fit the continuous zero inflated data, a Tweedie generalized mixed model (GLMM) with a log link function was applied using the package “glmmTMB” (Brooks et al., 2017). Covariates consisted of “time” (hours during the day), “number of VSTs” (as the number of different VSTs detected per hour), and “position” of recording in the vineyard (middle and border) as fixed factors. In addition, temperature, wind velocity, relative humidity, and air pressure were used as covariates representing environmental conditions. To account for the dependency among observations that were acquired during the same recording session and on the same site in the vineyard, “site” was used as a random factor. The variance inflation factors were calculated, and relative humidity was dropped because of its highly significant negative correlation with temperature (Zuur, Ieno & Elphick, 2010). After developing the full model (Eq. (1)), model assumptions and spatial dependency were verified (Zuur & Ieno, 2016). Akaike Information Criterion (AIC) was used for model selection, with further optimization of the covariates by backward selection (see Table S1).

(1) \begin{eqnarray*}S{A}_{\mathrm{ij}}Tweedie~(\mu ,{\sigma }^{\text{2}},\rho )\nonumber\\\displaystyle E(S{A}_{\mathrm{ij}})={\mu }_{\mathrm{ij}}\nonumber\\\displaystyle log({\mu }_{\mathrm{ij}})=Tim{e}_{\mathrm{ij}}+Number~of~VST{s}_{\mathrm{ij}}+Temperatur{e}_{\mathrm{ij}}+Wind~velocit{y}_{\mathrm{ij}}+fPositio{n}_{\mathrm{ij}}+Sit{e}_{\mathrm{i}}\nonumber\\\displaystyle Sit{e}_{\mathrm{i}}N(0,{\sigma }^{\text{2}})\end{eqnarray*}

where SA_ij_ is the j^th^ recording in Site_i_ (*i* = 1 to 26), and Site_i_ is the random intercept, which is assumed to be normally distributed with mean 0 and variance *σ*2.

The SA of *H. halys*, *S. titanus* and other unidentified insects was compared between periods of the day (morning, afternoon, and evening) using a Wilcoxon rank sum test.

To compare the daily SA of *S. titanus* in the vineyard and in semi-field conditions, the relationship between the hour average SA recorded in the vineyard (field) and in semi-field conditions, in the different recording periods, was assessed with Pearson correlation coefficients.

## Results

### Vibrational signal types and abiotic factors changes during the day

**Figure 2:**
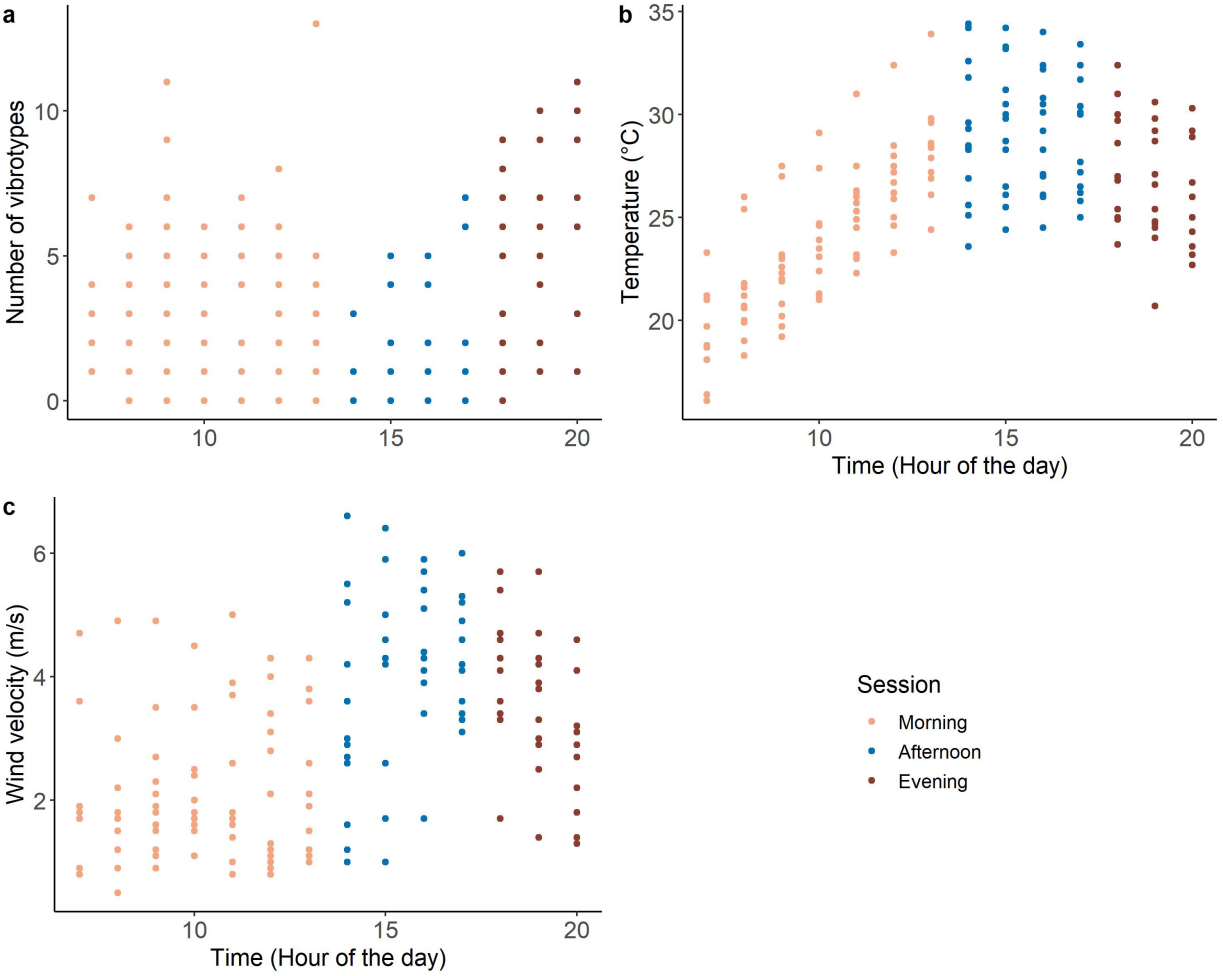
Variation of the covariates during the day: (A) number of VSTs; (B) temperature and (C) wind velocity. *n* = 173.

The morning (07:00–14:00) and evening (18:00–21:00) of the recording period were characterized by low temperature and low wind velocity, with the occurrence of many VSTs (range 0–13) (Fig. 2). While the afternoon (14:00-18:00) was characterized by high temperature and high wind velocity, with few VSTs (range 0-7) (Fig. 2).

### Signaling variation in the vineyard

**Figure 3:**
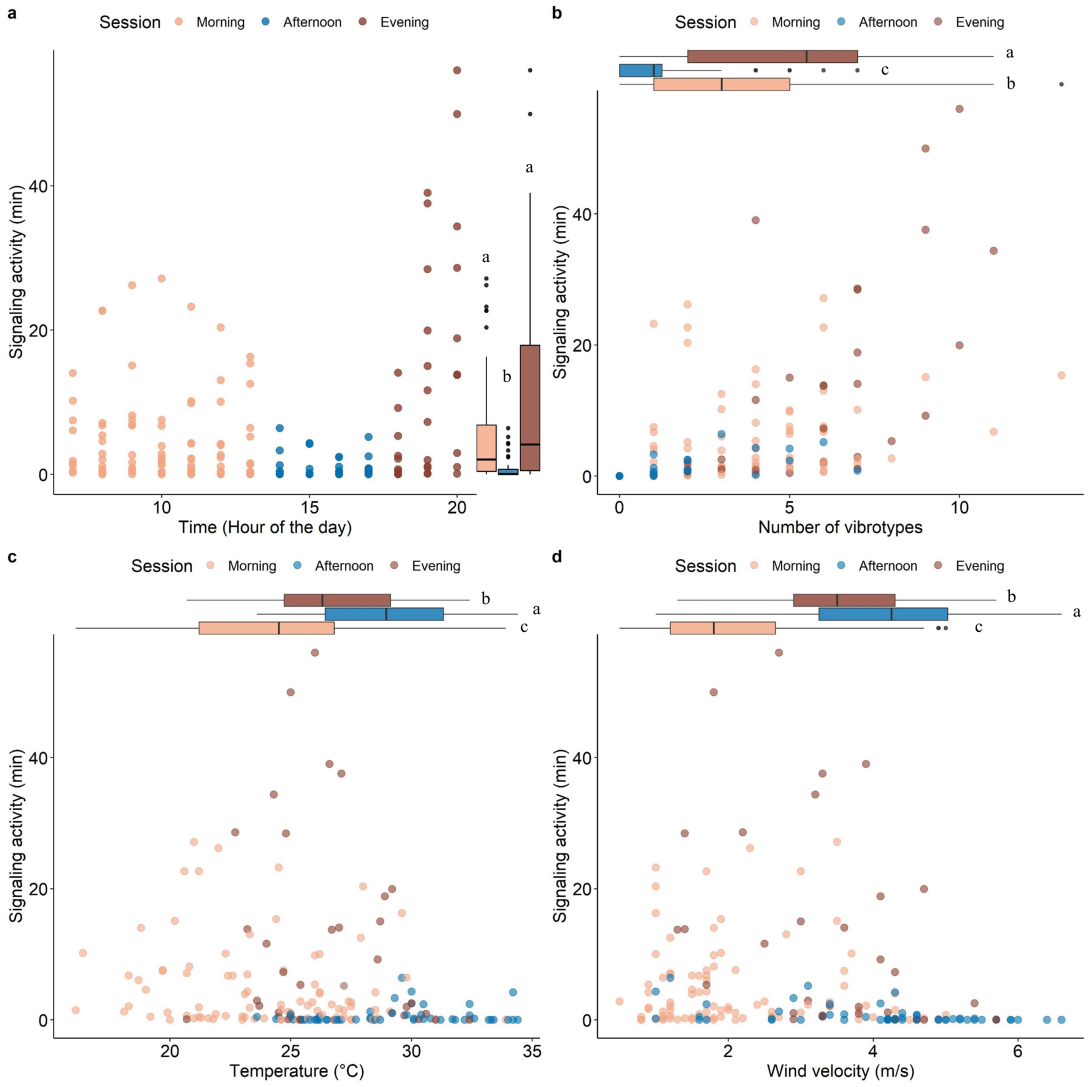
Scatterplots showing the effect of time of the day (A), the number of VSTs (B), temperature (C), and wind velocity (D) on the signaling activity. Boxplots on the right (A) or above (B, C, D) each scatterplot indicate the variation of the covariates between sessions, lowercase letters indicate significant differences (*p* < 0.05) after Wilcoxon rank sum test. *n* = 173.

Model validation, including spatial dependency (See Fig. S2), indicated no problems. The insect SA was highly variable throughout the day (range 0-54 min hour-1) and was significantly influenced by time, number of VSTs (3.08 ± 2.82), temperature (26.08 ± 3.93 °C), and wind velocity (2.97 ± 1.52 m/s) (Table 1). Fixed effects alone (marginal *R*^2^ = 0.42) and together with the random factor (conditional *R*^2^ = 0.66) had a high explanatory power in the used model. Most signaling occurred in the morning and in the evening, with a significant decrease in the afternoon (Fig. 3A). The signaling significantly increased with the number of VSTs (Fig. 3B). In total, 30 VSTs were detected with a maximum of 13 VSTs per hour (13:00–14:00). High SA was recorded in mild conditions of temperature (Fig. 3C; 20−30 °C) and wind velocity (Fig. 3D; <4 m s-1), while it strongly decreased at higher temperature (Fig. 3C; >30 °C) and wind velocity (Fig. 3D, >4 m s-1).

### Signaling of *H. halys* and *S. titanus* in the vineyard

**Figure 4:**
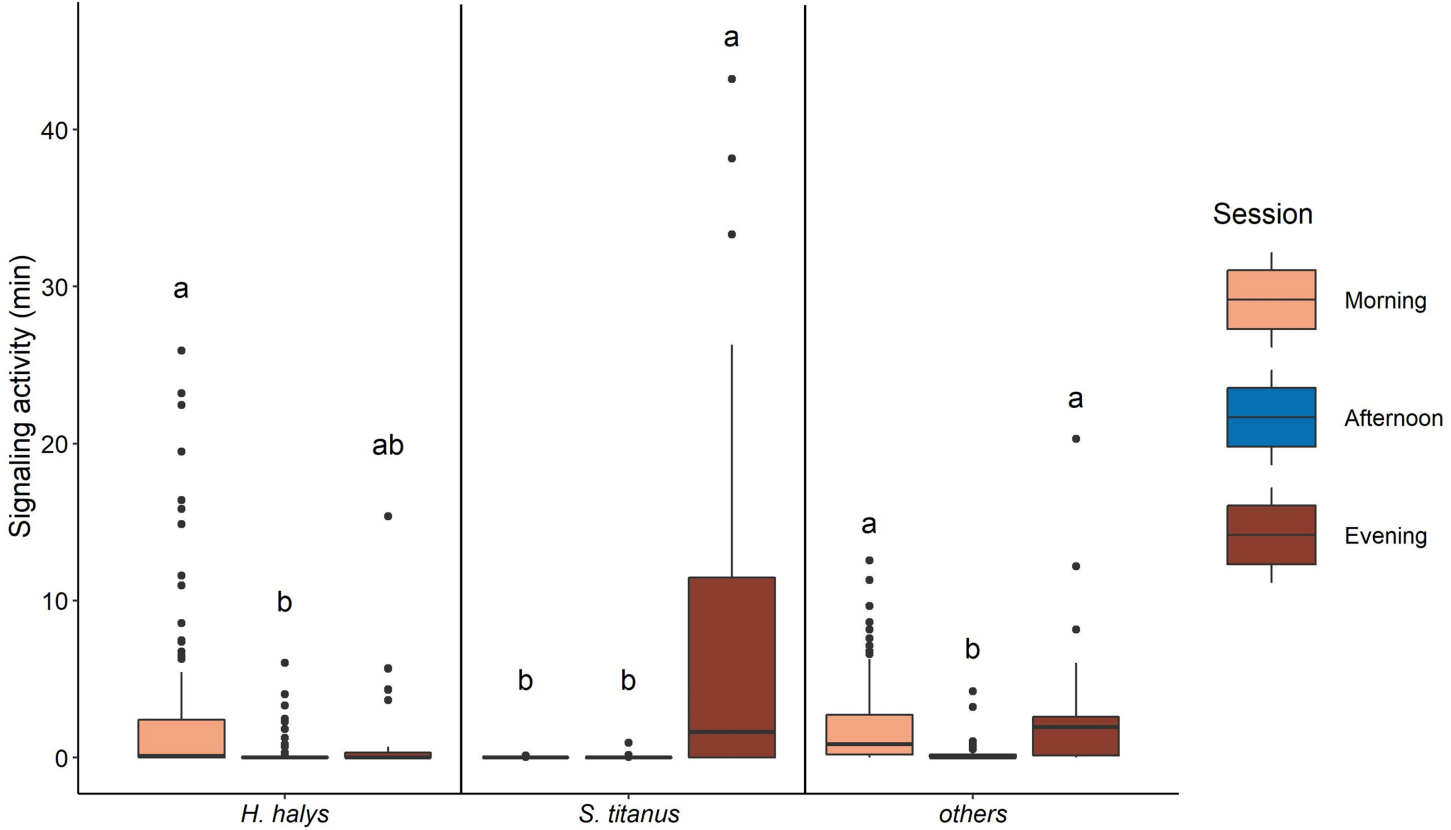
Signaling activity of *H. halys*, *S. titanus* and other, not identified, insects in the vineyard during the day, letters indicate significant differences (*p* < 0.05) between period of the day after Wilcoxon rank sum test.

The signaling of *H. halys* mostly occurred in the morning and evening, while very low SA was recorded in the afternoon (Fig. 4, Table 2, Fig. S3). Whereas SA of *S. titanus* was concentrated in the evening, and it was the main driver of the increase of the overall SA at that period of the day (Fig. 4, Table 2). Vibrational signals of *H. halys* were detected mostly in the recording sites situated on the borders of the vineyard while vibrational signals of *S. titanus* occurred in the middle (see Fig. S4). Vibrational signals of *E. vitis* were not detected in the recordings.

### Signaling of *S. titanus* in field and semi-field conditions

**Figure 5:**
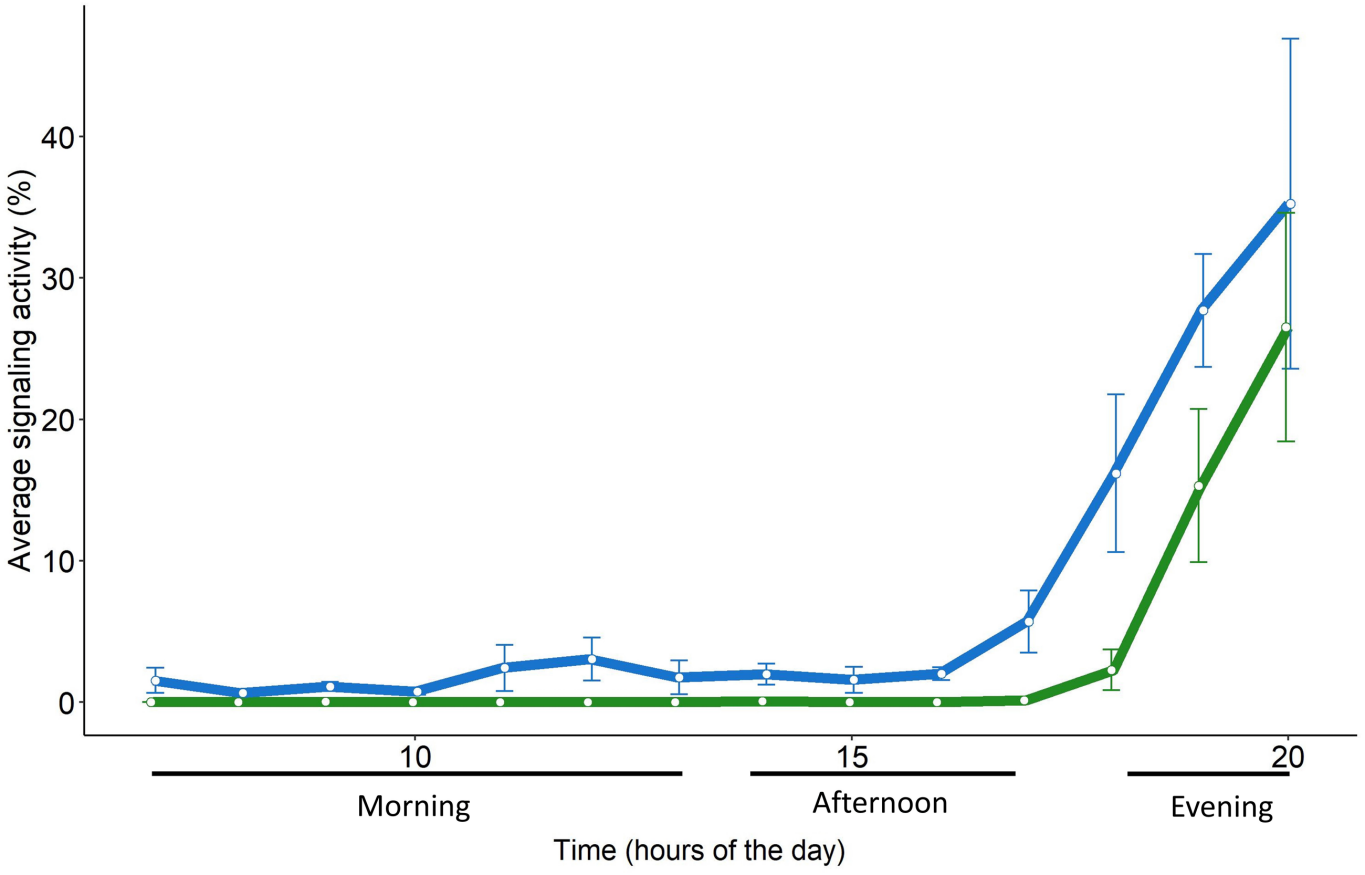
Daily pattern of the signaling activity of *S. titanus* in the vineyard (in green) and in semi-field conditions (in blue) presented as the average time insect spent signaling per hour. Standard error of the mean is shown with error bars.

The daily pattern of *S. titanus* SA recorded from the caged individuals was similar to that from the field, confirming that insects are mainly active in the evening (Fig. 5). However, in semi-field conditions vibrational signals of *S. titanus* were also occasionally recorded earlier in the day (from 11:00 to 13:00) and the peak of SA started from 17:00 while it started from 18:00 in field conditions. The correlation between the average signaling activity per hour recorded in the vineyard and semi-field (see Fig. S5) was significant only for the evening (*R* = 1, *p* = 0.048).

**Table 1:** Estimated regression parameters, standard errors, z ratio, and *p*-values from the Tweedie GLMM testing the effect of number of vibrotypes, time, temperature, wind velocity and position. Session was used as a random factor and the estimated value 0.973. The intercept corresponds to position: border.

	Estimate	Std. error	z value	*p*-value
Intercept	0.734	0.315	2.331	0.020
Time	0.624	0.165	3.779	<0.001
Number of VSTs	0.801	0.109	7.353	<0.001
Temperature	−0.510	0.138	−3.702	<0.001
Wind velocity	−0.515	0.138	−3.723	<0.001
Position: middle	−0.612	0.427	−1.433	0.152

## Discussion

The present study investigated the daily vibrational signaling of insects in field conditions in a vineyard and provided novel information of the effect of environmental conditions on their signaling activity. Although the total measured signaling activity resulted from vibrational signals of several different insect species, a common pattern of insect activity during a summer day in a vineyard was obtained. As expected, the signaling activity increased with the number of VSTs, which however are not necessarily associable to different species but in some cases could be part of the signal repertoire of the same species (Šturm et al., 2021).

Results showed that insect signaling in the vineyard changed according to time during the day which was mainly due to changes in temperature and wind velocity. In fact, most signaling occurred in the morning and evening when temperature and wind velocity were low, with the only exception of the early morning (7:00–08:00), when temperatures were below 20 °C. Cold conditions slow down the insect biological processes and for this reason many species can start calling only when a certain temperature is reached (Kingsolver, 2009). On the other hand, we consistently found low signaling activity in the afternoon when temperature and wind velocity reached the highest values. Our analysis indicates a significant effect of these two factors especially when certain combinations are met. A previous study showed the influence of these two parameters (even though their effect was investigated separately) on the mating behavior of *Nilaparvata lugens* (Hemiptera: Delphacidae) in controlled conditions (Ahmed et al., 2016). A similar daily pattern was also reported in field recordings of *Enchenopa binotata* (Hemiptera: Membraciade), where the daily vibrational signaling activity was associated with wind velocity (McNett, Luan & Cocroft, 2010). Our study shows for the first time, the combined effect of wind and temperature, deriving thresholds beyond which signaling activity is significantly reduced or even null. The reasons behind the cessation of the signaling activity in certain conditions, can be related to a strategy to increase the communication efficiency by reducing and preventing energy consumption and eavesdropping from antagonists. This strategy might be adopted to escape the signal masking by interferences from biotic (interspecific signals) or abiotic noise (wind and rain) (McNett, Luan & Cocroft, 2010; Tishechkin, 2013). Often, the highest signaling activity is recorded at intermediate temperature (*e.g.*, Leith, Jocson & Fowler-Finn, 2020; Macchiano et al., 2019) to meet optimal conditions of metabolism and to avoid excessive energetic costs (Erregger et al., 2017; Leith et al., 2021). An increase in ambient temperature can affect the characteristics of vibrational signals, by changing the temporal pattern but not the spectral structure, as it can increase the signal frequency and pulse rate in some species of spiders, bees, flies and planthoppers (Brandt, Kelley & Elias, 2018; Brandt, Rosenthal & Elias, 2020; Conrad, Stöcker & Ayasse, 2017; De Vrijer, 1984; Ritchie et al., 2001; Shimizu & Barth, 1996). For instance, changes in temperature induce differences in mating signals and therefore affects mate preferences (Brandt, Kelley & Elias, 2018; Jocson et al., 2019; Symes, Rodríguez & Höbel, 2017). The performance of the muscles involved in producing vibrations may depend on the thoracic temperature, which in turn depends on the ambient thermal conditions. The high temperature might increase the muscle contraction rate (Greenfield, 2002), which would result in high energy expenditure (Kuhelj et al., 2015). Although, relative humidity was not included in our analysis, it cannot be excluded that its effect is likewise important. Leafhoppers, as dominant species in the vineyard, prefer a microclimate of moderate temperature and relative humidity as they usually settle on the underside of grapevine leaves (Vidano, 1959). Results from earlier studies have indicated that wind can affect insect vibrational signaling, since it constitutes a major source of noise, disrupting arthropods that communicate by substrate-borne vibrations (Cocroft et al., 2014; Cocroft & Rodríguez, 2005; Tishechkin, 2007; Virant-Doberlet et al., 2014). Even at the peak of their activity, insects may reduce their signaling when exposed to wind gusts (Velilla et al., 2020). As a result, they may adjust the timing of their signaling according to wind velocity fluctuations or to the level of perceived noise (caused by the wind). Moreover, in conditions where strong wind occurs constantly during the day, insects may move to areas that are protected from the wind (Tishechkin, 2007; Tishechkin, 2013). High temperature and strong wind represented unfavorable conditions for insect signaling in the vineyard during a summer day and therefore these two abiotic factors should be considered when recording and evaluating vibrational signaling of insects in the field. Future studies should also compare the signaling activity of similar insect communities in areas characterized by different weather conditions and investigate whether wind and temperature could represent a selection pressure on the timing of insect vibrational signaling.

**Table 2:** The percentage of signaling activity of vineyard insects in each recording period.

Period	Insect	Percentage of signaling per Period
Morning	*H. halys*	57.91%
	*S. titanus*	0.14%
	others	41.95%
Afternoon	*H. halys*	61.38%
	*S. titanus*	3.26%
	others	35.36%
Evening	*H. halys*	9.38%
	*S. titanus*	68.26%
	others	21.91%

*Scaphoideus titanus*, in particular, concentrates the flight activity early in the morning and at dusk (Lessio & Alma, 2004), although laboratory trials conducted in the 24 h revealed that mating occurs mainly between 18.00 and 22.00 (Mazzoni et al., 2009). We found evidence that also in semi-field and field conditions the male calling activity remains concentrated in the evening, provided that there is the absence of strong wind (>4 m s^−1^) and the temperature is between approximately 18 and 30 °C. In semi-field conditions, sporadic signals of *S. titanus* were recorded also in the morning and one hour earlier in the evening in contrast to field conditions. Beside the fact that in semi-field conditions a higher number of individuals were consistently present on the same plant, it must not be excluded that in a cage the presence of several individuals in proximity may have triggered their interaction through accidental body contact or the detection of incidental signals derived by body movements (*e.g.*, grooming, walking) of other individuals. However, such differences are negligible when considering the general signaling pattern and peak of activity, indicating that vibroscape monitoring could reliably monitor the actual presence of *S. titanus* (and potentially of other species) in a vineyard.

Surprisingly, vibrational signals of *E. vitis* were not detected in our recordings. The signals could have been significantly attenuated before reaching the plant stem because of heterogeneity of the plant parts (Mazzoni et al., 2014) or they could have been masked by the background noise. This fact suggests that the vibroscape study should involve the use of different points on the same plant, for example by making short recording sessions of 30 min or 1 h or by using more recording instruments at the same time. In this regard, the use of accelerometers at high sensitivity and multichannel acquisition devices could be an optimal solution (Nieri et al., 2021). Another possible reason is the low amplitude of the vibrational signals that reduce the active space of *E. vitis* to a leaf area (R Nieri, 2021, pers. comm), while in the case of *S. titanus* (a bigger species: 5 mm *vs* 3 mm) the active space can include different leaves of the same plant and even leaves not in physical contact but sufficiently close (up to 7 mm) to the one where the insect is calling from (Eriksson et al., 2012). Therefore, when choosing the target species, size and signal amplitude should be taken into account. In this regard, we often detected the presence of *H. halys*, a relatively large species (up to 17 mm) (Hamilton et al., 2018) whose signal amplitude can be over 1 mm s^−1^ peak velocity (Polajnar et al., 2016b), that is 10 times higher than the signal amplitude of *S. titanus* (Eriksson et al., 2011). In the case of *H. halys*, a species abundant in the area (Zapponi et al., 2022), the signaling activity was mostly concentrated in the morning, although it never stopped during the day. This finding indicates an important difference between this species and *S. titanus*: the former remains sexually active most of the day and it is affected more by weather factors rather than having a precise daily cycle which is instead typical of the latter. It is still unclear if *H. halys* is sexually active also at night (as we did not see a clear peak of activity during the daytime) and this information would be important not only to fill an important biological knowledge gap, but also to better setup a strategy of pest management based on the use of vibrational traps. Such traps are currently the object of study and field testing (Caorsi et al., 2021; Zapponi et al., 2022) and we can anticipate their deployment in the field in the future. Since the emission of vibrational signals requires the use of electricity (that could be given by solar panels), it will be important to supply it especially when the insect sexual activity is higher.

The approach used in the present study can be regarded as the basis for a promising tool to monitor the presence and activity of vibrational signaling insects, in particular for leafhoppers, for which monitoring techniques are generally invasive (*e.g.*, sticky traps, sweeping nets) and not highly specific (affecting non-targets such as pollinators and natural enemies). In the absence of an updated reference library of vibrational signals, we cannot yet make precise assumptions about species composition, thus we cannot provide reliable ecological information in terms of biodiversity. Nevertheless, it would be possible in perspective to make comparisons between similar environments. In the case of vineyards or other orchards, beside the presence of key species, it seems to be feasible to quantify the occurrence of a complex of vibrational signals which could be used as a proxy of environmental quality. For example, as an instrument to measure the impact of pest management practices both on an insect community normally present in a certain site and on specific target species. In this regard, it becomes crucial to setup protocols of recording that aim at optimizing the signal recording efforts. For instance, enlarging the matrix of sampling points at plant and vineyard level, to detect insects that are characterized by a very narrow active space such as *E. vitis*. This would also increase the system sensitivity and fill the gap observed between field and semi-field tests. However, because moving from a plant to another during the recording sessions was challenging in our study, the use of light equipment such as accelerometers, would facilitate recording in multiple spots during shorter periods. A better option would be using fixed vibrational sensors deployed in a regular grid to record vibrational signals simultaneously and continuously, which could also enable evaluating the seasonal signaling variation and vibroscape composition, once a large library of vibrational signals is developed. A matrix of sensors may also point out the transition of insects through the agroecosystem such as the case of *H. halys* in the vineyard. Since vibrational signals are species and sex specific (Cocroft et al., 2006; Cocroft et al., 2014; Čokl & Virant-Doberlet, 2003; Virant-Doberlet, Cokl & Zorovic, 2006), and automated vibrational sensors have already been developed in biotremology (Korinšek, Tuma & Virant-Doberlet, 2019), their use in agroecosystems would allow the possibility to remotely detect and monitor insects without interfering with their behavior. For specific insect species, the set-up of sensors would be used to determine their daily signaling activity and target their monitoring at the peak time of their activity, for example in the evening in the case of *S. titanus*.

Furthermore, integrating a real time monitoring system with the association of the accelerometers to microcontrollers would allow the transmission of the information in real-time, providing information for decision support systems. Vibrational pest monitoring would help to optimize the schedule of interventions according to periods of actual occurrence and sexual activity of target insects or insect life stages.

## Conclusions

The present study provided evidence about the effect of abiotic factors on vibrational signaling of insects occurring on grapevine. Insects reduced their vibrational signaling during time of the day where high wind velocity and temperatures were recorded. This is only a first step in the direction of a larger use of biotremology techniques for new aims. For example, we consider the effect of anthropogenic vibrational noise an important element to integrate in future studies, in order to understand how the human presence may affect the insect vibrational activity. Similar to ecoacoustics, where acoustic diversity indices have been correlated with traditional diversity indices (Gage, Napoletano & Cooper, 2001) and used as a method of biodiversity assessment (Sueur et al., 2012; Sueur et al., 2008), we think that biotremology could be applied for studies to monitor and compare the biodiversity in agroecosystems.

##  Supplemental Information

10.7717/peerj.14143/supp-1 Supplemental Information 1 Supplementary Figures

10.7717/peerj.14143/supp-2 Supplemental Information 2 Raw data The data of the field measurements in terms of signaling duration (in minutes) and corresponding parameters included in the model and the Fig. 1 and 2.
The field measurements of signaling duration of the identified insects used for Fig. 3.
The data of Scaphoideus titanus signaling duration in field and semi-field measurements that were used for Fig. 4.

## Additional Information and Declarations

### Data Availability

The following information was supplied regarding data availability: The raw measurements are available in the Supplemental Files.
