# Supplementary material for: Extending the vibroscape to agroecosystems: investigating the influence of abiotic factors and monitoring insect vibrational signaling
Source: PeerJ. 2022 Nov 17;10:e14143. doi: 10.7717/peerj.14143 (PMC9676016; doi:10.7717/peerj.14143)
Supplement: Supplemental Information 1 [file peerj-10-14143-s001.docx]

Extending the vibroscape to agroecosystems: investigating the influence of abiotic factors and monitoring insect vibrational signaling

# **Imane Akassou1,2,*, Livia Zapponi2, Vincenzo Verrastro3, Marco Ciolli1,4, and Valerio Mazzoni 2**

1DICAM Department of Civil, Environmental and Mechanical Engineering, University of Trento, Trento, Italy

2Research and Innovation Centre, Fondazione Edmund Mach, San Michele all’Adige (TN), Italy

3CIHEAM–IAMB - International Centre for Advanced Mediterranean Agronomic Studies, Bari, Italy

2C3A, Centre Agriculture Food Environment, University of Trento, San Michele all’Adige (TN), Italy

*imane.akassou@unitn.it

**
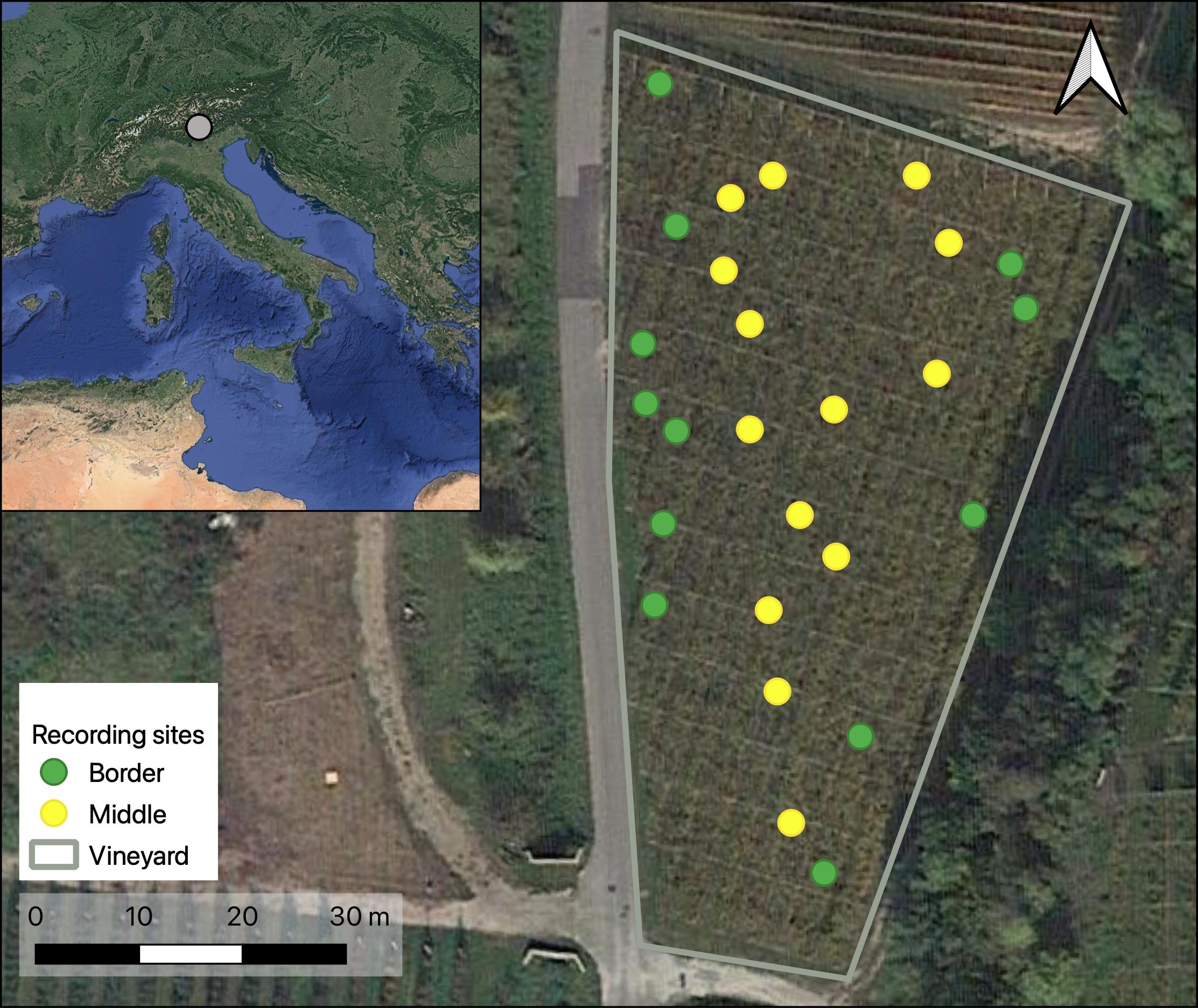
**

**Figure S1:** Map of the recording sites in the vineyard. Each point represents the position of the plant where vibrational signals were recorded during a recording session.

**
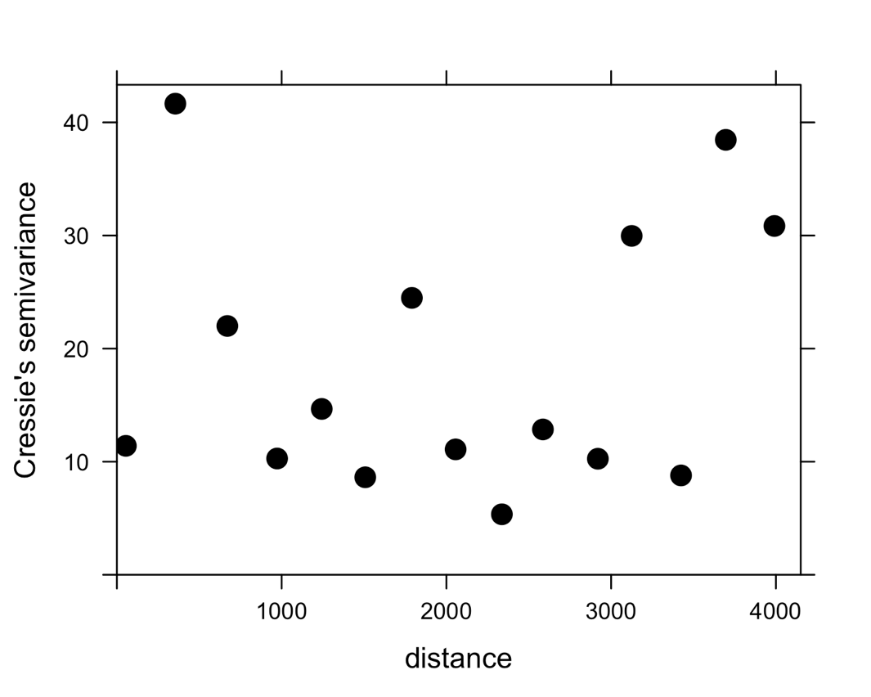
**

**Figure S2**: Semi-variogram for the residuals from the final model (Equation 1).


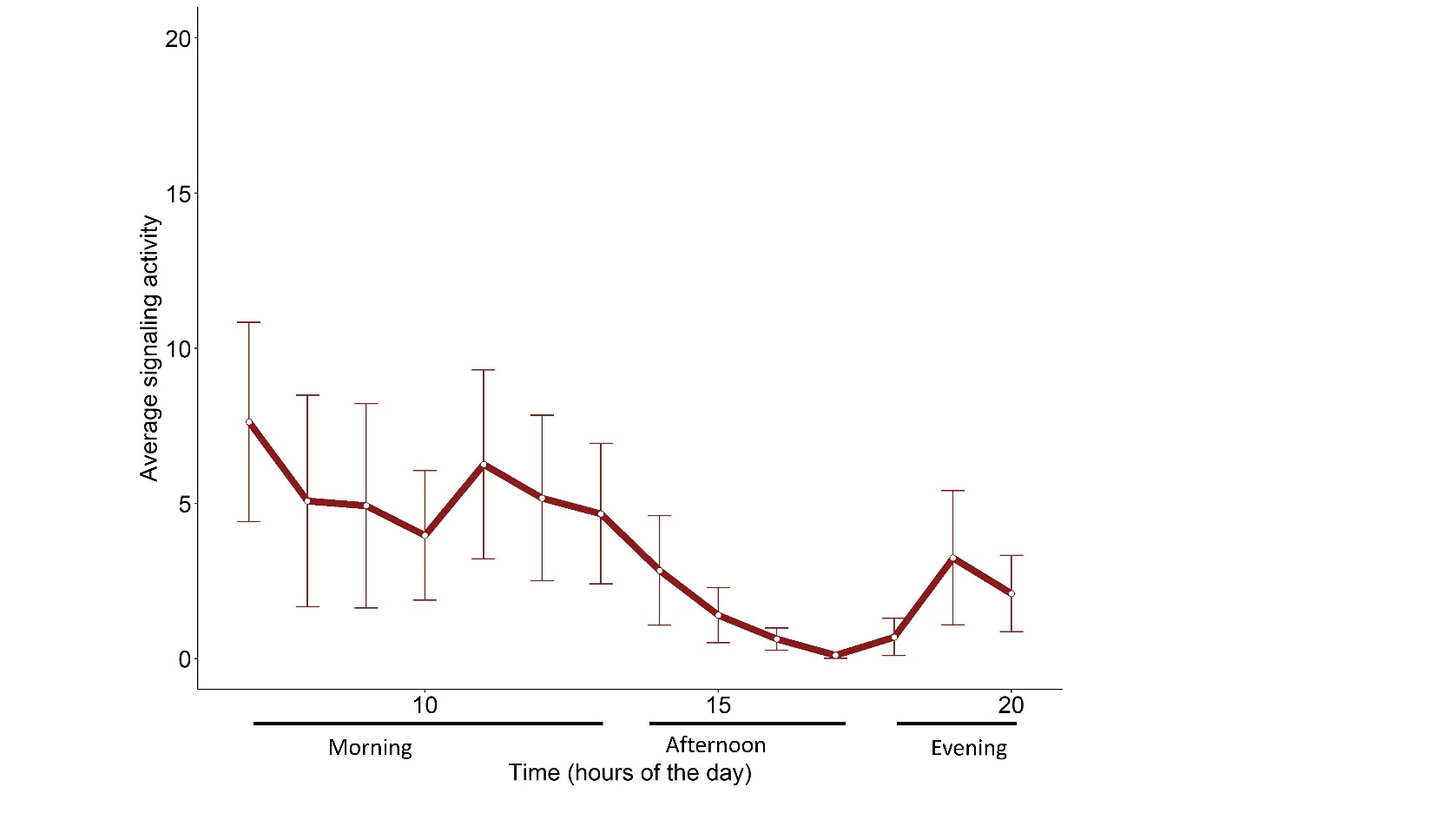


**Figure S3:** Daily pattern of the signaling activity of H. halys in the vineyard presented as the average time insects spent signaling per hour. Standard error of the mean is shown with error bars.


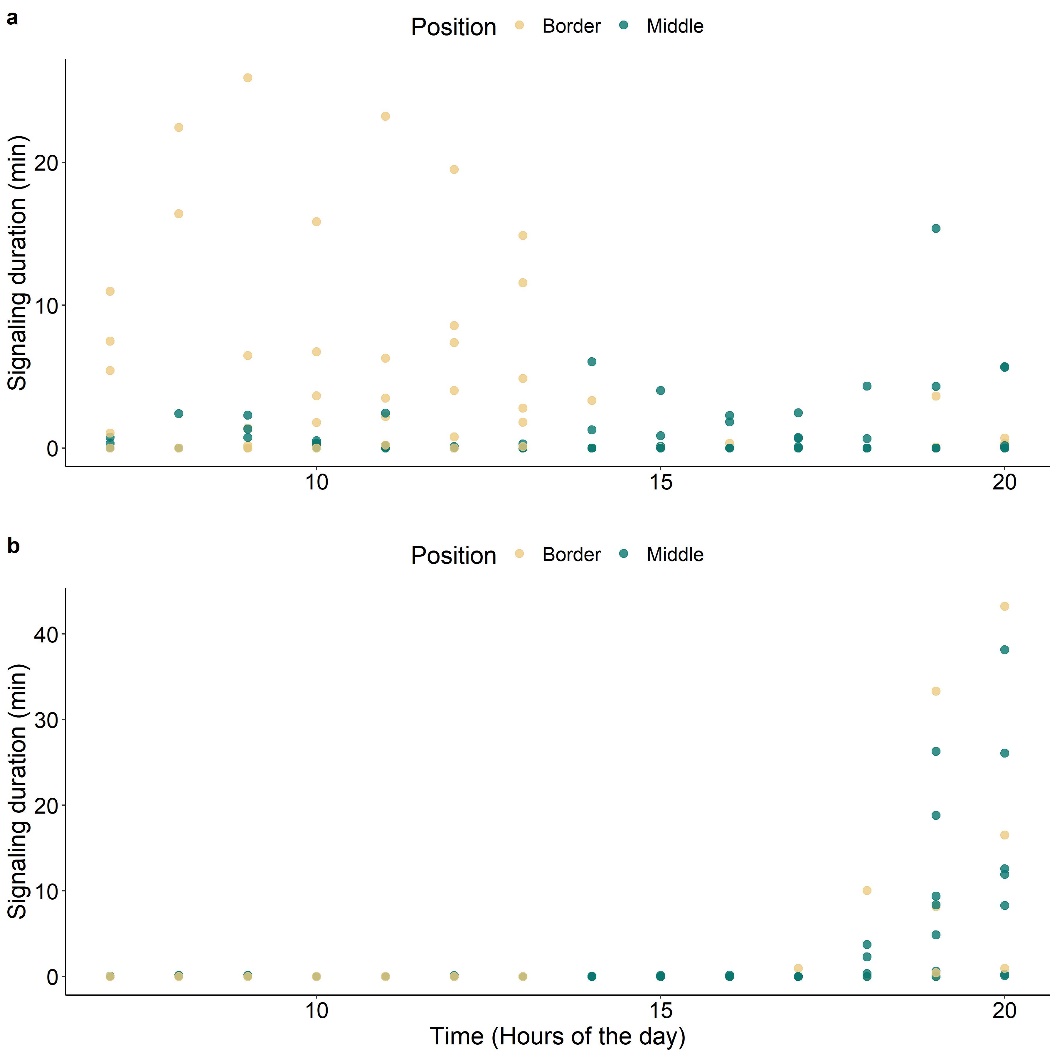


**Figure S4:** Scatterplots showing the vibrational signaling activity of *H. halys* (**a**) and *S. titanus* (**b**) during that day, according to position of recording in the vineyard as border (yellow) or middle (blue).


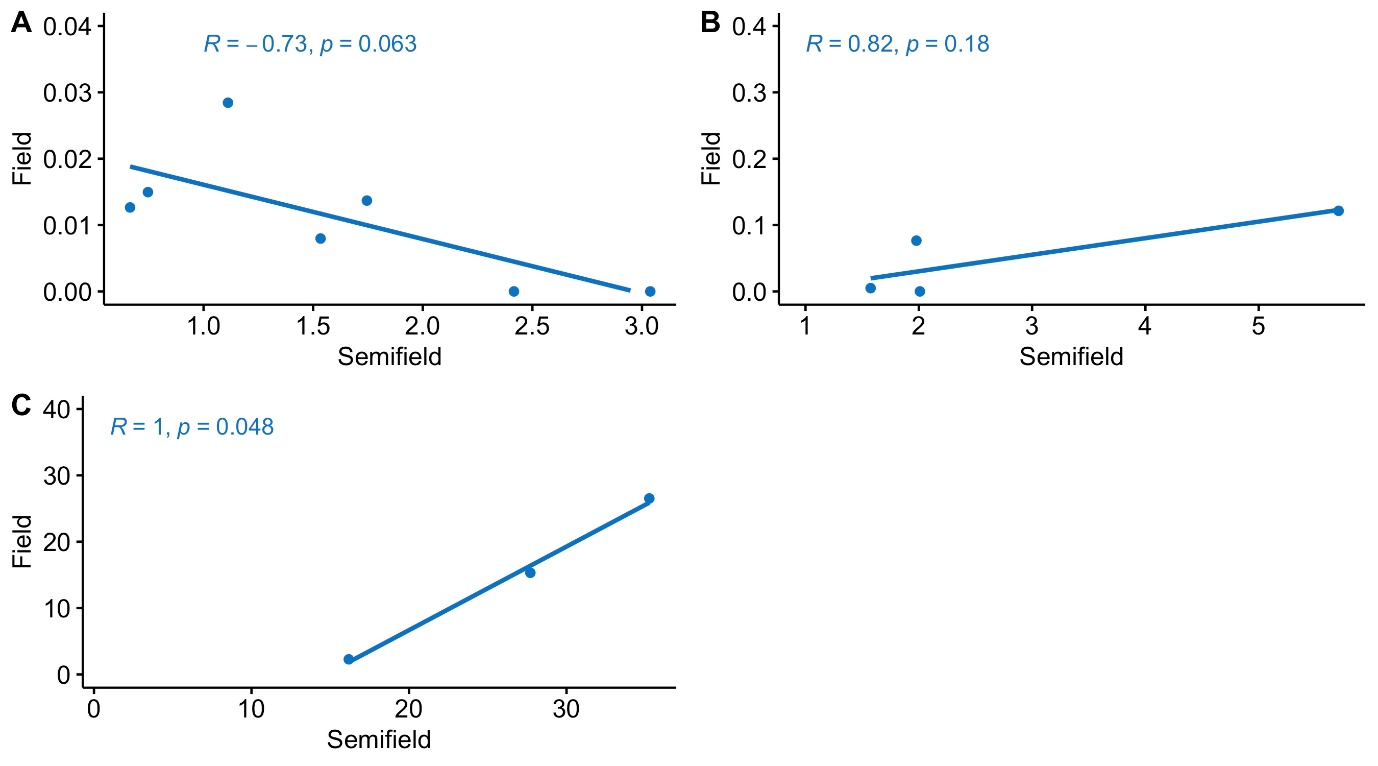


**Figure S5.** Scatterplots with regression lines for the correlation between the hour average signaling activity of S. titanus recorded in the vineyard (field) and in semi-field conditions: **A**. morning, **B**. afternoon and **C**. evening.

| Model formulation | AIC |
| --- | --- |
| SA ~ Temperature * Position * Wind velocity + Number of vibrotypes +   Air pressure + Time | 742.71 |
| SA ~ Temperature * Position * Wind velocity + Number of vibrotypes +   Time | 741.37 |
| SA ~ Temperature + Position + Wind velocity + Number of vibrotypes +   Air pressure + Time | 741.32 |
| SA ~ Temperature + Position + Wind velocity + Number of vibrotypes +   Time | 739.95 |

**Table S1:** Akaike information criterion (AIC) for the candidate models.
